# Performance Portrait Method: An Intelligent PID Controller Design Based on a Database of Relevant Systems Behaviors

**DOI:** 10.3390/s22103753

**Published:** 2022-05-14

**Authors:** Mikulas Huba, Damir Vrancic

**Affiliations:** 1 Institute of Automotive Mechatronics, Faculty of Electrical Engineering and Information Technology, Slovak University of Technology in Bratislava, SK-812 19 Bratislava, Slovakia; 2 Department of Computer Automation and Control, J. Stefan Institute, SI-1000 Ljubljana, Slovenia; damir.vrancic@ijs.si

**Keywords:** multiple real dominant pole method, performance portrait, PID control, disturbance observer, ultra-local models

## Abstract

The article deals with a computer-supported design of optimal and robust proportional-integral-derivative controllers with two degrees of freedom (2DoF PID) for a double integrator plus dead-time (DIPDT) process model. The particular design steps are discussed in terms of intelligent use of all available information extracted from a database of control tracking and disturbance rejection step responses, assessed by means of speed and shape-related performance measures of the process input and output signals, and denoted as a performance portrait (PP). In the first step, the performance portrait method (PPM) is used as a verifier, for whether the pilot analytical design of the parallel 2DoF PID controller did not omit practically interesting settings and shows that the optimality analysis can easily be extended to the series 2DoF PID controller. This is important as an explicit observer of equivalent input disturbances based on steady-state input values of ultra-local DIPDT models, while the parallel PID controller, allowing faster transient responses, needs an additional low-pass filter when reconstructed equivalent disturbances are required. Next, the design efficiency and conciseness in analyzing the effects of different loop parameters on changing the optimal processes are illustrated by an iterative use of PPM, enabled by the visualization of the dependence between the closed-loop performance and the shapes of the control signals. The main contributions of the paper are the introduction of PPM as an intelligent method for controller tuning that mimics an expert with sufficient experience to select the most appropriate solution based on a database of known solutions. In doing so, the analysis in this paper reveals new, previously undiscovered dimensions of PID control design.

## 1. Introduction

The ever-increasing possibilities of computer technology allow an ever-increasing part of the processes related to the automatic control of systems to be carried out using programmable devices. Their design usually attempts to emulate important characteristics of human intelligence, leading to a discipline called intelligent control.

The effort to mimic the helmsman’s action needed to control ships was at the birth of proportional-integral-derivative (PID) regulators [1]. With the development of computer technology, it was also further enhanced to fuzzy control [2,3,4,5,6]. Today, intelligent control means, in addition to fuzzy control, a much broader class of control techniques that use various artificial intelligence (AI) computing approaches like neural networks [7,8,9,10,11,12], Bayesian probability [13,14], particle swarm optimization [15,16,17], machine learning, reinforcement learning [18,19,20], evolutionary computation, or genetic algorithms [21,22,23,24]. Of course, this calculation is not definitive and can be expected to grow further, with the aim to propose new solutions (as in, e.g., [25,26]) satisfying the continuously increasing new requirements of practice.

Intelligent control methods are successfully promoted, especially in areas where conventional control design is associated with the need to solve complex problems, such as, e.g., arising in nonlinear systems with incomplete and inadequate representation and under incomplete specifications of how to do this in an uncertain environment toward a not definitely specified control goal [4,5]. Their use also tends to reflect the personality of the designer, who prefers intuitive and heuristic design methods and returns to the analytical model-based approach only to verify and validate his intuitive results.

A large amount of work in the field of AI has already been devoted to optimizing the settings of PI and PID controllers. However, tied to the intrinsic limitations of this type of control, it has not always led to significant progress in the achieved performance. The reasons could be partially caused by the incomplete understanding of the functionality of these controllers. Just recently, they have been discussed as structures for system stabilization with an explicit or implicit disturbance observer (DOB) for the reconstruction of the input disturbance from steady-state input related to ultra-local integral models of controlled systems [27,28]. Although this type of DOB represented by a first-order low-pass filter is interesting due to its exceptional simplicity, it inherently places significant limitations on the dynamics of the reconstruction processes and on the acceptable settings of the controllers. In borderline, but not entirely rare, cases, these intrinsic limitations of PID control can finally lead to the abuse of the concept of intelligent control, when the adjective “intelligent” is rather a sign of the pursuit of a cheap and effective attribute. Ultimately, such approaches can lead to pejorative attitudes to misleading overly simplified “intelligent” designs.

The presented work brings a more consistent emphasis on two aspects of PID controller design—system modeling using ultra-local integral models and possible DOB-based PID interpretation—which belong to the basic indebtedness of traditional methods of automatic control and their innovative modifications used within intelligent control. Whereas the use of ultra-local models is today frequently preferred in such areas as model-free control (MFC) [29,30], or active disturbance rejection control (ADRC) [31,32], in terms of use in the field of PID control (despite frequent use, as in [33,34,35]), this is a neglected issue. Furthermore, the DOB-based view on PID control represents a relatively new and not yet sufficiently and systematically explored topic [27,28].

In addition, the paper develops another new dimension of an intelligent approach to the design of optimal and robust controllers based on the performance portrait method (PPM). After several initial steps [36,37], the PPM was fully developed, especially in the works [38,39]. It could be simply stated that this contribution represents an extension of the work dealing with optimal and robust control design of systems approximated by integrator plus dead-time (IPDT) models using 2DoF PI controllers [38] to the case of controlling DIPDT models by 2DoF PID controllers. However, such a formulation of the focus of the work is only very indicative because the control of DIPDT models brings a number of new moments that have influenced the scenario of work at many points.

With respect to this, the paper is structured as follows. A brief overview of the plant modeling from the point of view of positional control of autonomous vehicles is discussed in Section 2, together with the basic problems of an analytical 2DoF PD and PID controller design.

Section 3 discusses the basic limitations of an analytical design of optimal 2DoF PID control and introduces the ideal shapes of transient responses, together with the related performance measures enabling us to evaluate deviations of achieved transients from their ideal wave-forms. Section 4 deals with the introduction of the performance portrait (PP) and its use in the performance portrait method (PPM), allowing optimal nominal parallel and series PID controller tuning. Contrary to [28], it offers an extension of PPM with the possibility of weighting the emphasis on the resulting behavior in terms of setpoint tracking and disturbance rejection while also considering the optimization of the reference setpoint signal weighting by optimal settings of pre-filter parameters.

Discussion of the main results of the paper in Section 5, together with their summary in the Conclusions, show that due to strong limitations of the up-to-now available mathematical apparatus, just the intelligent control based on the PPM is able to analyze and exploit all (even up to now hidden) dimensions of PID control.

## 2. PD and PID Controller Schemes for Analysis and Implementation

In order to be able to use illustrative physical interpretations of the considered steps when working with PPM and also to stress the differences with respect to the traditional approaches to PID control typical, e.g., in process control, let us assume the application of the method for controlling the position of vehicles with one degree of freedom of movement. Due to the existing analogies of the description of dynamic systems, the method can, of course, also be fully used for the control of any other dynamic systems, for the reliable stabilization of which the proportional-derivative (PD) controller must be used. As the main specific feature of the chosen domain of interpretation, we will highlight the fact that in terms of reference setpoint tracking, the need for smooth monotonic setpoint step responses without over-regulation will be emphasized.

### 2.1. Double-Integrator and Double-Integrator-Plus-Dead-Time Models

**Figure 1:**
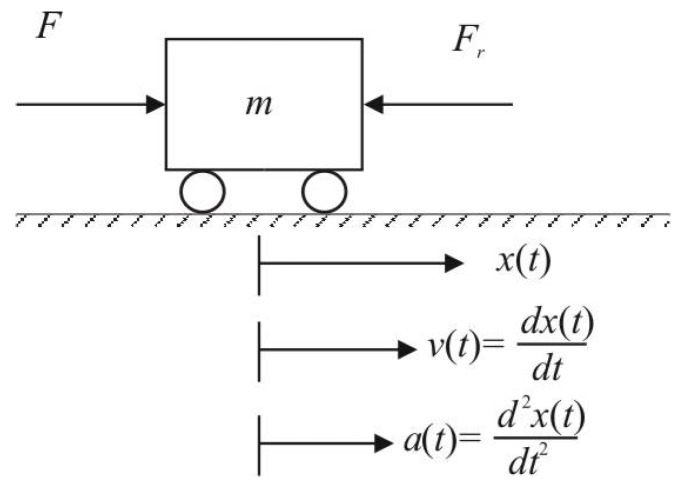
Description of an autonomous vehicle movement in one dimension.

Due to the forces changing nonlinearly during a vehicle motion, whereby several system parameters are not fully known and can vary in time, control of a moving body can be treated by several possible approaches to nonlinear uncertain systems. All the possible approaches can be unified by application of Newton’s second law when the vehicle acceleration is proportional to the resulting force applied (F=ma). When we leave the designations typical of physics in Figure 1 and instead denote the position of the vehicle by the variable *x*, its velocity as $\dot{x}$ and acceleration as $\ddot{x}$, and after introducing the gain $K_{s}=1/m$ instead of the mass *m*, the system equation can be written in the form:

(1) $$\ddot{x}=K_{s}\left(u_{r}+d_{i}-F_{r}\left(x,\dot{x}\right)\right).$$

Thereby, $u_{r}\equiv F$ corresponds to the system’s manipulated variable (control signal), $F_{r}\left(x,\;\dot{x}\right)$ represents the resulting force acting against the control signal (consisting of friction and gravitational forces), and the input disturbance $d_{i}$ summarizes the influence of external or internal forces not taken into account by the considered model.

When considering repeated movements in a known environment and with a known load, it probably makes sense to apply nonlinear methods to the control design as exact linearization [40], or to consider linearization around the selected fixed operating point, replacing nonlinear dependencies $F_{r}\left(x,\;\dot{x}\right)$ with “local” linear approximations obtained, e.g., by the Taylor series expansion. However, when moving in an unknown environment, it is usually impossible to work on the control design with linear or nonlinear approximations $F_{r}\left(x,\;\dot{x}\right)$ depending on *x* and $\dot{x}$. There is nothing left but to merge the unknown value $F_{r}\left(x_{0},\;{\dot{x}}_{0}\right)$ in the current state $x={\left(x_{0},\;{\dot{x}}_{0}\right)}^{t}$ with the external disturbance $d_{i}$ into an equivalent disturbance:

(2) $$d_{e}=d_{i}+F_{r}\left(x_{0},\;{\dot{x}}_{0}\right)$$

as typically used in ADRC.

In case of output disturbances $d_{o}$, the output equation can be written as:

(3) $$y=x+d_{o}.$$

As for the output disturbance, it can be, for example, an inaccuracy in the location of the vehicle or the drift of ships by sea currents. However, with regard to the inertia of the movement, the output disturbances of a jumping nature are certainly not possible, and therefore we will limit ourselves to considering the jump changes of the required position and the input disturbance (load) and consider y=x.

In addition, when neglecting the possible control saturation by considering $u\left(t\right)=u_{r}\left(t\right)$ and taking into account that all other delays arising in the transmission and processing of information, in the actuators, filters, and sensors can be denoted as a model dead-time $T_{m}$, the double integrator plus dead time (DIPDT) model [28,41] can be written as:

(4) $$\begin{array}{c} F\left(s\right)=\frac{Y(s)}{U(s)}=\frac{K_{s}}{s^{2}}e^{-T_{m}s} \end{array}$$

In the case of a non-measurable output derivative, its value has to be reconstructed by a derivation combined with a binomial low-pass filter $Q_{n}\left(s\right)$:

(5) $$\begin{array}{c} Q_{n}\left(s\right)=\frac{1}{{\left(T_{f}s+1\right)}^{n}};\;n\geq 1 \end{array}$$

Thereby, the sampling period $T_{s}$ used for implementation must be chosen to fulfill $T_{s}<<T_{f}$. The total loop dead-time $T_{d}$ must include, in addition to an estimate of the loop delay $T_{m}$, an equivalent filter delay estimate $T_{e}$:

(6) $$T_{d}=T_{m}+T_{e};\;T_{e}=nNT_{f},$$

where $T_{e}$ (as proposed in [42]) approximates the filter dynamics in terms of a dead time that can simply be added to the total dead time $T_{d}$, where the coefficient *N* can be given by values in the range from N=0.5 (equivalence based on “the half rule”) to N=1 (equivalence based on “the average residence time”).

### 2.2. Stabilizing PD Controller Tuning by the Triple Real Dominant Pole Method

Today, even people without an education in automatic control, but with the experience of a car driver, know that to stabilize the movement of the vehicle, its position, speed, load, and the driver’s reaction time must be taken into account. Thus, to control the DIPDT system (4) with a constant input disturbance $d_{i}$, a proportional-derivative (PD) controller expressed with the help of the Laplace transform as $C_{s}\left(s\right)=K_{p}\left(1+T_{D}s\right)$ and supplemented by a disturbance feedforward counteracting the equivalent disturbances with the equation:

(7) $$\begin{array}{c} u=K_{p}\left(1+T_{D}s\right)\left(w-y\right)-{\hat{d}}_{e} \end{array}$$

has to be used, where *y* denotes the actual and *w* the reference setpoint output values. Here, ${\hat{d}}_{e}$ represents a measurement, or estimate of the equivalent disturbance $d_{e}=d_{i}+F_{r}\left(x_{0}\right)$. Analysis of an optimal controller tuning for a DIPDT system by the triple real dominant pole method can be found in [41,43]. In [44], the PD controller has been proposed by modification of PI control for an IPDT system based on the relative time-delay margin. Furthermore, ref. [45] starts with the modification of PI control for an IPDT system, whereby the PD controller design is based on pole placement by using the Lambert W function.

DIPDT model stabilization by an ideal PD controller:

(8) $$U\left(s\right)=\left(K_{p}+K_{d}s\right)E\left(s\right)$$

having at its input the (tracking) control error:

(9) E(s)=W(s)−Y(s)

yields the closed loop transfer function:

(10) $$F_{wy}\left(s\right)=\frac{Y(s)}{W(s)}=\frac{K_{s}\left(K_{d}s+K_{p}\right)}{e^{T_{d}s}s^{2}+K_{s}\left(K_{p}+K_{d}s\right)}$$

Its characteristic quasi-polynomial:

(11) $$A_{PD}\left(s\right)=e^{T_{d}s}s^{2}+K_{s}\left(K_{p}+K_{d}s\right)$$

has a triple real pole $s_{o}$, when the loop parameters fulfill the requirements:

(12) $$\begin{array}{c} {\left[A_{PD}\left(s\right);\;\frac{dA_{PD}\left(s\right)}{ds};\;\frac{dA_{PD}^{2}\left(s\right)}{ds^{2}}\right]}_{s=s_{o}}=0 \end{array}$$

i.e., when:

(13) $$s_{o}=\epsilon /T_{d}=-0.5858/T_{d};\;T_{o}=-1/s_{o}=1.7071T_{d}$$

Here, $T_{o}$ denotes the time constant associated with the real pole $s_{o}$. As pointed out by one of the anonymous reviewers, according to [46], for the given closed-loop model, the maximum pole multiplicity is three. At the same time, to determine two unknown controller parameters and the position of the corresponding multiple pole, one has to solve a system (12) with minimally three solutions.

Although the analytical design of controllers of systems with delays and the transcendent nature of the mathematical description is associated with limitations, it will help to clarify several other possibilities of numerical solutions of the problem. The fastest non-oscillatory responses corresponding to a triple-real-dominant-pole (TRDP) $s_{o}=-0.5858/T_{d}$, which yields $K_{p}=0.079/\left(K_{s}T_{d}^{2}\right);\;K_{d}=0.461/\left(K_{s}T_{d}\right)$, can finally be shown to be guaranteed by dimensionless controller parameters:

(14) $$\begin{array}{c} \kappa_{o}=K_{p}K_{s}T_{d}^{2}=0.079;\;\delta_{o}=K_{d}K_{s}T_{d}=0.461;\;\tau_{D}=\delta_{o}/\kappa_{o}=5.8284;\\ p_{o}=s_{o}T_{d}=-0.5858. \end{array}$$

Due to the zero of $F_{wy}$ in (10), the corresponding setpoint step responses typically have overshoot. For its elimination, a pre-filter must be added to the PD controller (8):

(15) $$\begin{array}{c} F_{p}\left(s\right)=\frac{1+bs}{1+T_{D}s};\;\;T_{D}=\frac{K_{d}}{K_{p}};\;\;b=T_{o}, \end{array}$$

Its denominator cancels the numerator zero $-1/T_{D}$. The numerator coefficient *b* can be determined to cancel one of the triple poles of the closed-loop $s_{o}=-1/T_{o}$.

### 2.3. From the PD Controller to the Series and Parallel PID Controller Design

**Figure 2:**
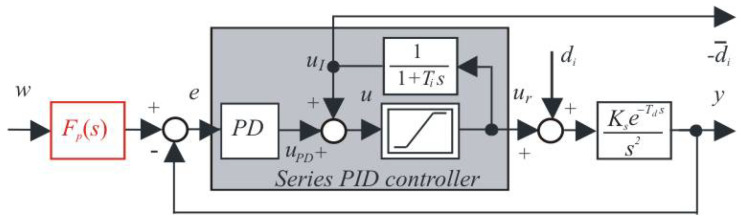
Creating 2DOF series PID controller from PD controller for DIPDT plant.

In addition to Minorsky’s work [1], which influenced the development of the simplest systems control theory and the development of relevant terminology by designing the three-term control concept, there was also a practical branch of research in which pneumatic-based controllers prevailed for a long time [47]. Although there has been significantly less information published in this area (in terms of patent protection), over time, elements of an intelligent approach that sought to emulate and replace the work of experts can also be traced. Thus, proportional-integration (PI) controllers were first created, in which the need to set the additional controller output signal (offset) required to achieve zero permanent control error under the influence of constant disturbances was replaced by the introduction of positive feedback from the controller output [27,28]. This created the controllers, originally called “automatic reset”. Their essence can be explained by the observation that in integral systems, the steady state-output of the controller is equal to the negative value of the input disturbance $d_{i}$. Because a counteracting signal must be added to the controller output to compensate for the constant $d_{i}$ acting on the plant input, a double inversion of the sign gives positive feedback. To measure the steady-state values of the controller output $u_{r}$ (see Figure 2), it was sufficient to take a given signal using a low-pass filter with a time constant $T_{i}$ substantially longer than the time constant of transients in the circuit with the stabilizing controller (denoted in previous section as $T_{o}$). Relatively short transients caused by input step changes affect the output of such a filter only negligibly, and the controller steady-state output values dominate in it.

At this moment, it is necessary to stress that only when neglecting the constraints put on the control action in Figure 2 (only in the proportional control band), can the corresponding series PID controller be described by the transfer function:

(16) $$C_{s}\left(s\right)=\frac{U(s)}{E(s)}=K_{cs}\frac{\left(1+sT_{is}\right)\left(1+T_{Ds}s\right)}{sT_{is}}$$

where the controller parameters considered with the indices “*s*” are: $K_{c}$ the controller gain, $T_{i}$ the integral, and $T_{D}$ the derivative time constant.

In such a situation, it is then also possible to get the parallel PID controller:

(17) $$C_{p}\left(s\right)=\frac{U(s)}{E(s)}=K_{cp}\frac{1+T_{ip}s+T_{ip}T_{Dp}s^{2}}{T_{ip}s}$$

with parameters denoted with the index “*p*”. To get in both structures the same controller transfer function, it must hold:

(18) $$C\left(s\right)=\frac{U(s)}{E(s)}=K_{cs}\frac{\left(1+sT_{is}\right)\left(1+T_{Ds}s\right)}{sT_{is}}=K_{cp}\frac{1+T_{ip}s+T_{ip}T_{Dp}s^{2}}{T_{ip}s}$$

Whereas the re-calculation from a series to a parallel controller is always possible according to:

(19) $$\begin{array}{c} T_{ip}=T_{is}+T_{Ds};\;K_{cp}=K_{cs}\frac{T_{is}+T_{Ds}}{T_{is}};\;T_{Dp}=\frac{T_{is}T_{Ds}}{T_{is}+T_{Ds}} \end{array}$$

the replacement of a parallel PID controller by a series one according to:

(20) $$\begin{array}{c} T_{is}=\left[T_{ip}\pm \sqrt{\left(T_{ip}^{2}-4T_{ip}T_{Dp}\right)}\right]/2\\ K_{cs}=K_{cp}\left[T_{ip}\pm \sqrt{\left(T_{ip}^{2}-4T_{ip}T_{Dp}\right)}\right]/\left(2T_{ip}\right)=\\ =K_{cp}\left[0.5\pm \sqrt{\left(0.25-T_{Dp}/T_{ip}\right)}\right]\\ T_{Ds}=\left[T_{ip}\mp \sqrt{\left(T_{ip}^{2}-4T_{ip}T_{Dp}\right)}\right]/2 \end{array}$$

is only possible for $T_{ip}\geq 4T_{Dp}$.

### 2.4. Optimal Parallel PID Tuning by the Quadruple Real Dominant Pole Method

In [48], the PID controller design has been based on SIMC design [49,50]. In [33], it has been modified, based on optimization using the exact gradient method. In [44], the controller optimization was based on the dead-time margin.

Next, the analytical controller tuning will be based on the quadruple real dominant pole (QRDP) method [28,41,42]. If, for simplicity, we omit the index “*p*” from the parameter designation, application of the parallel PID controller (17) leads to the closed-loop transfer functions:

(21) $$\begin{array}{c} F_{wy}\left(s\right)=\frac{Y(s)}{W(s)}=\frac{K_{c}K_{s}\left(1+T_{i}s+T_{i}T_{D}s^{2}\right)}{T_{i}s^{3}e^{T_{d}s}+K_{c}K_{s}\left(1+T_{i}s+T_{i}T_{D}s^{2}\right)}\\ F_{iy}\left(s\right)=\frac{Y(s)}{D_{i}\left(s\right)}=\frac{K_{s}T_{i}s}{T_{i}s^{3}e^{T_{d}s}+K_{c}K_{s}\left(1+T_{i}s+T_{i}T_{D}s^{2}\right)} \end{array}$$

Similar to above, from the characteristic quasi-polynomial:

(22) $$P\left(s\right)=T_{i}s^{3}e^{T_{d}s}+K_{c}K_{s}\left(1+T_{i}s+T_{i}T_{D}s^{2}\right)$$

with four unknown parameters yields the requirement of a quadruple real dominant pole (QRDP) $s_{o}$, which requires the conditions:

(23) $${\left[P\left(s\right);\;\;\frac{dP\left(s\right)}{ds};\;\;\frac{dP^{2}\left(s\right)}{ds^{2}};\;\frac{d^{3}P\left(s\right)}{ds^{3}}\right]}_{s=s_{o}}=0$$

to be met, the dimensionless parameters of the parallel PID controller:

(24) $$\begin{array}{c} p_{o}=s_{o}T_{d}=-0.416;\;\tau_{o}=-1/p_{o}=2.405;\\ \kappa_{p}=K_{cp}K_{s}T_{d}^{2}=0.1248;\;\delta_{p}=K_{dp}K_{s}T_{d}=0.5045;\;\eta_{p}=K_{ip}K_{s}T_{d}=0.0121;\\ \tau_{ip}=T_{ip}/T_{d}=10.324;\;\tau_{Dp}=T_{Dp}/T_{d}=4.043. \end{array}$$

Overshooting of the setpoint step responses due to zeros of $F_{wy}$ in (21) can now be eliminated by a pre-filter:

(25) $$\begin{array}{c} F_{p}\left(s\right)=\frac{1+bs+cs^{2}}{1+T_{ip}s\left(1+T_{Dp}s\right)} \end{array}$$

with the trivial numerator tuning:

(26) $$b_{0}=c_{0}=0.$$

The setpoint responses can be accelerated by cancelling a single dominant pole $s_{o}$, and even more by cancelling two dominant poles $s_{o}^{2}$, when:

(27) $$c_{1}=0,\;b_{1}=T_{o},\;\mathrm{or}\;c_{2}=T_{o}^{2},\;b_{2}=2T_{o};\;\;T_{o}=-1/s_{o}=2.405T_{d}$$

## 3. Basic Limitations of Analytical Tuning of PD and PID Controller

If we want to evaluate the basic pros and cons of the above analytical setup of PID controllers for the DIPDT system, we must first state the performance measures appropriate to the problem and its applications in (vehicle) control.

### 3.1. Evaluation of the Speed of Transient Response

For the tracking error (9) defined in the time domain as e(t)=w(t)−y(t), the speed of transients can be evaluated in terms of the integral of the absolute error (IAE):

(28) $$IAE=\int_{0}^{\infty }\left|e(t)\right|dt.$$

For the unit setpoint step responses and $d_{i}=d_{o}=0$ it will be denoted as $IAE_{w}$. For $w\left(t\right)=0,d_{o}=0$ and unit input disturbance (load) step as $IAE_{i}$. Some works (for example, [33,44]) used in derivation of Pareto-optimal (PO) PID controller with maximal sensitivity constraints in the cost function:

(29) $$J=s_{w}\frac{IAE_{o}}{IAE_{o,min}}+s_{i}\frac{IAE_{i}}{IAE_{i,min}};\;s_{w}+s_{i}=1$$

where $IAE_{o}$ corresponds to unit step responses of the output disturbance $d_{o}$. However, they do not give examples of applications with the possibility of step changes of $d_{o}$. Of course, when applied to vessel control (as in [44]), output disturbances caused by drift due to sea currents can be expected. However, in no case will these be jump disturbances, and it is, therefore, questionable whether such optimization will lead to concise results. Similarly, when driving autonomous vehicles, output disturbances may occur due to inaccurate GPS location. However, such disturbances will be rather random in nature, and it will be more appropriate to classify them as measurement noise. In the following, therefore, the cost function:

(30) $$J=s_{w}\frac{IAE_{w}}{IAE_{w,min}}+s_{i}\frac{IAE_{i}}{IAE_{i,min}};\;s_{w}+s_{i}=1$$

similar to [38,51] will be used to evaluate the optimality of control.

Unit setpoint step responses lead under the TRDP PD controller (14) to optimal IAE values:

(31) $$IAE_{w}=4.1213T_{d}.$$

When considering the parallel PID controller, depending on the prefilter (25) numerator order, the QRDP PID tuning (24) yields IAE values corresponding to unit setpoint and disturbance steps:

(32) $$IAE_{w0}=10.323T_{d};\;IAE_{w1}=7.918T_{d};\;IAE_{w2}=5.513T_{d};\;IAE_{i}=82.728T_{d}^{3}.$$

Significant differences in the optimal values $IAE_{w}$ and $IAE_{i}$ of the controller’s analytical design show why standardization using minimum achievable values $IAE_{w,min}$ and $IAE_{i,min}$ is necessary when optimizing general designs with an emphasis on setpoint tracking or disturbance rejection. They also document that, with the introduction of I-action, it is possible to compensate for the disturbances acting, but at the cost of prolonging the transients (in the case given by $IAE_{w2}/IAE_{w}$ by 5.513/4.1213=1.34 times, 1.92 times for $IAE_{w1}$, and by 2.5 times for $IAE_{w0}$) with the current paradoxical increase in proportional gain ($K_{c}/K_{p}=0.1248/0.079=1.58$ times).

When someone thinks about ways to improve the performance of the loops using a PID controller, it is also important to remember that:

**Remark** **1** (Fundamental restrictions on PID controller integral time constant). *The integral time constant $T_{i}=10.324T_{d}$ (24) of the QRDP PID controller must be much longer than the dominant time constant $T_{o}=1.707T_{d}$ of transients stabilized by the TRDP PD controller (13).*

Detailed research based on the analysis of several other optimal settings of PI and PID controllers [27,28] has shown that this fundamental limitation of the disturbance reconstruction rate and thus the transient speed resulting from the nature of the disturbance observer used (reconstruction from steady-state controller output values) cannot be removed even using artificial intelligence methods.

**Remark** **2** (The inability to achieve QRDP optimal responses by a series PID controller). *It follows from the transformation relations (20) for the conversion of the series controller parameters from the parallel PID parameters that due to:*

(33) $$\sqrt{(0.25-T_{Dp}/T_{ip}}=0.25-4.043/10.324=-0.1416<0$$

*it is impossible to achieve QRDP optimal responses by a series PID controller with real parameters. Instead, optimal series PID controllers always have (see, for example, [33]):*

(34) $$\tau_{is}=\tau_{Ds}$$

*However, such a selection requires that their value be appropriately determined and the value of $K_{cs}$ added.*

### 3.2. Evaluating the Excessive Controller Effort

In application of PID control, a high attention has to be devoted to measurement noise impact, which in combination with not appropriate controller tuning can lead to an unacceptable excessive controller effort. In [33,44,48], it has been evaluated in terms of total variation:

(35) $$TV\left(u\right)=\sum_{i=0}^{\infty }\left|u_{i+1}-u_{i}\right|$$

Although TV(u) plays an important role in circuit assessment, the identification of this measure with excessive controller effort is incorrect because the total amount of absolute increments also includes useful control signal changes used to achieve the desired output change [50]. In other words, reaching the “ideal” value of TV(u)=0 would mean that the output of the DIPDT system if it was initially in a steady state, would not change at all during the transient.

Although the value of TV (35) is related to excessive control efforts, it does not in itself have an immediate physical or mathematical interpretation. However, appropriate TV modifications can be useful in evaluating the deviations of the time responses from optimal shapes based on the concept of monotonicity. Monotonic output variable changes mean that all its increments have contributed to the output change from an initial value $y_{0}$ to the final value $y_{\infty }$ without causing some counteractive movement. Thus, the output signal samples $y_{i}$ viewed with the sampling period $T_{s}$ yield a measure [38]:

(36) $$TV_{0}\left(y\right)=\sum_{i=0}^{\infty }\left|y_{i+1}-y_{i}\right|-\left|y_{\infty }-y_{0}\right|$$

that can be interpreted as a deviation from monotonicity. $TV_{0}\left(y\right)>0$ signalizes a non-monotonic transient with some increments not contributing to the requested change, e.g., for a smooth response with a single overshoot amplitude Δ, $TV_{0}\left(y\right)=2\Delta$. Therefore, $TV_{0}\left(y\right)$ finds application mainly in the evaluation of setpoint step responses at the system output.

The situation will be more complicated when evaluating the output waveforms after a disturbance step. Due to the delayed response of the controller, which occurs as a result of to the transport delay of the system, the deviation of the output from the setpoint will increase in the first phase of the input disturbance step coming after steady state. When the response of the controller finally manifests itself, ideally the output of the system monotonically returns to the desired value: the output thus consists of two monotonic sections. Such a course is hereinafter referred to as one pulse (1P) shape. The deviation of the real course from its ideal 1P shape is calculated by summing the deviations from the monotonicity on these two individual sections according to:

(37) $$TV_{1}\left(y\right)=\sum_{i}\left|y_{i+1}-y_{i}\right|-\left|2y_{m}-y_{\infty }-y_{0}\right|$$

where $y_{m}\notin \left(y_{0},y_{\infty }\right)$ lying outside the interval formed by the initial and final output values $y_{0}$ and $y_{\infty }$ represents the point separating the monotonic sections of the 1P response.

Feldbaum’s principle of time-optimal control of a vehicle described by the second-order differential equation expressed the need for two intervals with constrained control signal alternating individual limits more than half a century ago [52]. It is true that in the literature focused on smoother PID controllers, Feldbaum’s theorem has been practically forgotten (with exception of few authors, as in [53,54]). However, the requirement to account for active control pulses applies not only to relaying rectangular control interventions, but also to their smoother modifications, in which case some of the limit values may not even be reached. Especially in the field of vehicle motion control, it is certainly not possible to optimize their movement by minimizing the value of TV, but it must be adjusted for the values of useful interventions needed to accelerate and brake the vehicle. Thus, for a double integrator [37,38], the optimal shape of the control signal $u\left(t\right),t\in \left[0^{-},\infty \right)$ associated with an ideal monotonic setpoint changes and 1P disturbance responses of the output (given by the inverse plant dynamics) will be specified by two extreme points $u_{m1}$, $u_{m2}$ occurring at times $t_{1},t_{2}\in \left(0,\infty \right)$, lying between the initial and final values $u_{0}$ and $u_{\infty }$ and satisfying the condition:

(38) $$\left(u_{m1}-u_{\infty }\right)\left(u_{m2}-u_{\infty }\right)<0.$$

They form three monotonic control intervals of the two-pulse (2P) optimal control signal shape and the excessive control effort (summing the deviations from monotonicity over these three intervals) has to be evaluated according to:

(39) $${TV}_{2}\left(u\right)=\sum_{i}\left(\left|u_{i+1}-u_{i}\right|\right)-\left|2u_{m1}-2u_{m2}+\left(u_{\infty }-u_{0}\right)sign\left(u_{m1}-u_{\infty }\right)\right|$$

**Figure 3:**
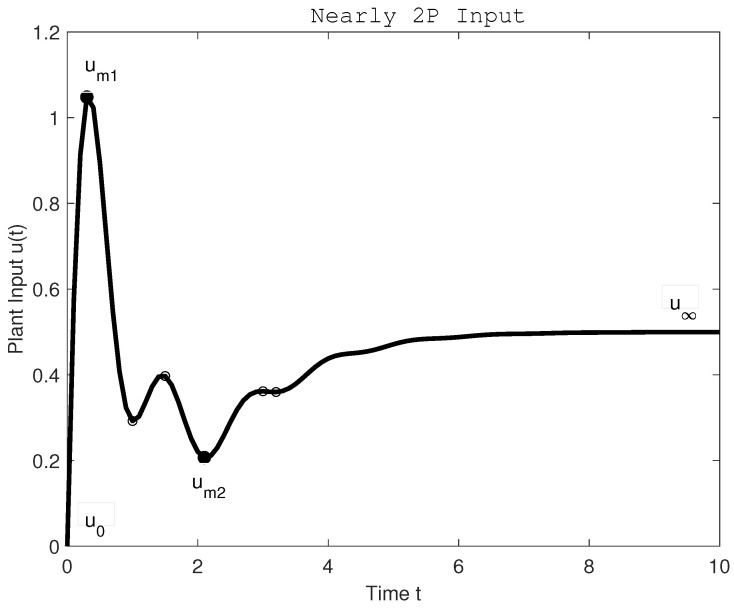
A “useful” control signal contribution of a more complex plant input (controller output) calculated as: $TV_{2}\left(u\right)=u_{m1}-u_{0}+u_{m1}-u_{m2}+u_{\infty }-u_{m2}=2u_{m1}-2u_{m2}+u_{\infty }-u_{0}$.

It means that for more complex controller output shapes (as, for example, in Figure 3), the total variations (35) has to be decreased by a “useful” contribution term combining the acceleration-deceleration amplitudes $2(u_{m1}-u_{m2})$ with the total control signal change $u_{\infty }-u_{0}$ considered with the polarity depending on the first amplitude value with respect to the final steady-state control signal.

### 3.3. Example 1: Performance Evaluation of PD and PID Controllers

To illustrate the properties of derived TRDP PD and QRDP PID controllers, consider their evaluation using the proposed performance measures and comparison with the design of the PD controller according to [45].

For the plant model (4), implementation filter (5), and the delay equivalence (6) with parameters:

(40) $$K_{m}=K_{s}=1;\;T_{m}=1;\;N=1;\;n=1;\;T_{f}=T_{m}/4;\;T_{sim}=30;$$

it will be considered:

1. The QRDP PID controller (24) ($K_{p}=0.08;\;K_{d}=0.4043;\;T_{D}=5.0537;\;T_{i}=12.905;$ $T_{o}=3.0062$) and the prefilter (25) with the numerator tuning $c_{1}=0,\;b_{1}=T_{o}$ (QRDP);
2. The TRDP PD controller (14) ($K_{p}=0.0506;\;K_{d}=0.3688;\;T_{D}=7.2943;\;T_{o}=2.1339$) and the prefilter (15) with the numerator tuning $b=T_{o}$ (TRDPb);
3. The TRDP PD controller (14) and the prefilter (15) with the numerator tuning b=0 (TRDP0);
4. The PD controller according to [45] with parameters $K_{p}=0.12705/\left(KsT_{d}^{2}\right)=0.0813;$ $K_{d}=0.53228/\left(KsT_{d}\right)=0.4258$, the prefilter (15), $T_{D}=K_{d}/K_{p}=5.2369$ with the numerator tuning b=0 (GJ).

**Figure 4:**
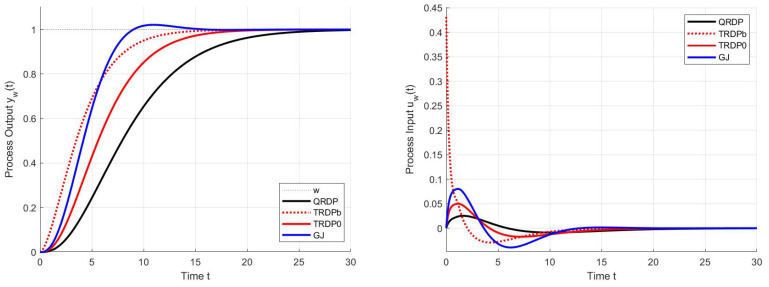
Setpoint step responses of the QRDP PID controller (24) with the prefilter (25), $c_{1}=0,$$b_{1}=T_{o}$, TRDP PD controller (14) with the prefilter (15), $b=T_{0}$ (TRD Pb) and b=0 (TRDP0) and the PD controller according to Gerov and Jovanovic [45] with the prefilter (15), b=0 (GJ).

Simulation with the step $T_{s}=0.001$ yields the setpoint step responses in Figure 4 with the performance measures in Table 1.

In terms of $IAE_{w}$ (Table 1), the fastest transients apparently correspond to the TRDPb controller. However, due to the zero relative degree of the prefilter, there is a discontinuity of the control action variable at the beginning. The undesirably high peak in *u* can be removed by using a saturation block, without significantly slowing down the transient that occurs in the case of TRDP0. Similar problems would arise when ingesting TRDP PID with a zero relative degree prefilter. Thanks to the reconstruction of disturbances in the TRDP PID from steady-state values of the controller output, this controller also yields the slowest transients. However, all responses with controllers set by the multiple real pole method are characteristic with a minimum number of monotonic sections at the input and output of the system, which corresponds to the zero values of $TV_{0}\left(y_{w}\right)$ and $TV_{2}\left(u_{w}\right)$.

When using the controller GJ according to [45], the output response is already over-regulated even when using the pre-filter (15) with b=0, which is signaled by the non-zero value $TV_{0}\left(y_{w}\right)$. The output overshooting $\Delta y=0.021<TV_{0}\left(y_{w}\right)/2$ then also increases the overall controller activity signalized by the $TV_{2}\left(u_{w}\right)>0$ value.

## 4. Performance Portrait Method

Examining all possible meaningful solutions to a problem before deciding which one best meets the defined criteria is one of the basic aspects of “intelligent” problem solving. It is the basis of a method called brainstorming, but also of such legally established procedures as public procurement. Verification of assumed meaningful solutions is also an approach that is very popular in the design of optimal control by practitioners who prefer experimentation over a theoretically justified design. They return to the theoretical arguments only when they already know the optimal solution and use the theoretical justification to support its adoption.

The performance portrait method (PPM) [28,36,37,38] assumes verification of loop properties at all possible relevant controller settings. For the DIPDT models, such meaningful intervals of optimal controller parameters were analytically investigated by selecting different groupings of real dominant poles in [28]. After choosing the appropriate quantization of particular dimensionless variables, which should be fine enough to capture all the important specifics of the problem under consideration, it enabled one to limit the control design to a grid of points and thus to spare the time and energy. The finer the quantization is choosen, the higher the total number of points at which one has to verify the properties of the system, either by simulation or real-time control. By concise approximation (modeling) of initial systems and work with dimensionless parameters (as in (24)), the applicability of information obtained by system analysis on a given grid can be extended to a significantly wider set of systems.

When analyzing closed loop properties at the controller parameters corresponding to the selected tuning point, several performance measures are evaluated, such as $IAE_{w}$, $IAE_{i}$, $TV_{0}\left(y_{w}\right)$, $TV_{1}\left(y_{i}\right)$, $TV_{2}\left(u_{w}\right)$, $TV_{2}\left(u_{i}\right)$, corresponding to the steps of the reference variable *w* and the load disturbance $d_{i}$. This list is, of course, not exhaustive and may be extended or modified as necessary (for example, by the values of the sensitivity functions $M_{s}$ and $M_{t}$ preferred by many authors, maximum overshoot values, settling times, etc.). As we showed in the previous example, the amplitude of the maximum overshoot can also be estimated from the value of $TV_{0}\left(y_{w}\right)$. Since the values of $M_{s}$ and $M_{t}$ are important in relation to the limits of stability, but with higher demands on positioning accuracy and the shape of transients, they do not give as concise information as deviations from monotonicity and will not be used in this contribution.

**Definition** **1** (Performance Portrait (PP) and Performance Portrait Method (PPM)).*The information about the process is obtained, evaluated, and stored using computer technology. If necessary, it can be step-wise extended or reduced and reused for optimal and robust design of control circuits.*

*The performance portrait method (PPM) represents a digitization of controller settings inspired by the behavior of practitioners who first verify everything by experimenting. It is a reflection of four types of learning styles [55] in the field of controller design and by its nature falls within the trends of Industry 4.0.*

Furthermore, if someone refers to the performance portrait method (PPM) as a “brute force” approach [34], it should be noted that traditional methods of optimizing controller parameters are much closer to that name. With PPM in general, there is no problem with convergence to the optimal solution, while the search for optimums can be repeated with minimal effort indefinitely, with the choice of new criteria of optimality (or using their various combinations).

### 4.1. Example 2: Generating PP of the Parallel PID Controller

To facilitate the possibility of repeating the next procedure to potential applicants, the generation of PP will be explained using simulation of the loop with a 2DoF parallel PID controller with an additional disturbance observer. Based on the experience of [28] and after adding another dimension needed to determine the appropriate prefilter coefficient *b*, it follows as a suitable choice:

(41) $$\begin{array}{c} \kappa \in [0.05,0.35];\;\Delta \kappa =0.01;\;\delta \in [0.15,1.05];\;\Delta \delta =0.03;\\ \tau_{i}\in \left[6,22\right];\;\Delta \tau_{i}=2;\;b\in \left[0.1,0.9\right];\;\Delta b=0.1. \end{array}$$

**Figure 5:**
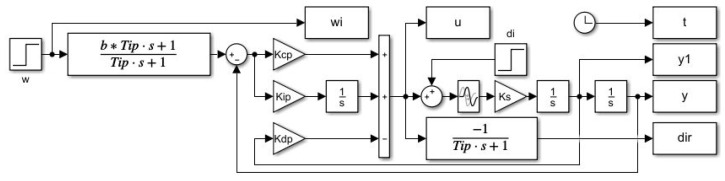
Matlab/Simulink simulation scheme of the parallel PID controller used to generate PP (41) with the prefilter $F_{p}\left(s\right)=\left(1+bT_{ip}s\right)/\left(1+T_{ip}s\right)$ (42) and an additional low-pass filter used for disturbance reconstruction.

In order to be able to generate PP independently of the choice of the derivative action filter needed for the parallel PID controller implementation, a state-space scheme with available output derivative will be used (Figure 5) with the prefilter:

(42) $$F_{p}\left(s\right)=\frac{bT_{i}s+1}{T_{i}s+1}$$

deleting the zero of $F_{wy}\left(s\right)$:

(43) $$F_{wy}\left(s\right)=\frac{K_{c}K_{s}\left(T_{i}s+1\right)}{T_{i}s^{2}\left(se^{T_{d}s}+K_{c}K_{s}T_{i}\right)+K_{c}K_{s}\left(1+T_{i}s\right)}$$

The PP will be generated by simulating the unit setpoint and input disturbance step responses corresponding to the grid parameters (41). For each grid point, the performance measures $IAE_{w},\;IAE_{i},\;TV_{2}\left(u_{w}\right),\;TV_{2}\left(u_{i}\right),\;TV_{0}\left(y_{w}\right),\;TV_{1}\left(y_{i}\right)$ will be stored in the corresponding performance measures matrices.

The simulations have been carried out in a total of 31×31×9×9+31×31×9= 86,490 points. The duration of the PP calculation on a standard PC was 37 h. Because the calculation of performance measures at individual points is independent of each other, the whole problem can be advantageously parallelized. Of course, in the traditional evaluation of experiments by practitioners, it would not be realistic to work with such a high number of experiments and process characteristics using different performance measures. Thus, in terms of the detailed information considered in the controller design, PPM multiplies the capabilities of human experts.

In the next process of using PPM, we determine whether there is a point (or in the case of uncertain systems a compact group of points) in which a closed circuit would meet the specified limits on the values of dimensionless parameters. These must be chosen so that the requirements for the real system with specific values of the DIPDT model parameters are ultimately met.

### 4.2. Example 3: Optimal Nominal Tuning of the Parallel PID Controller

Choice of the “optimal” nominal solution will be based on weighted IAE values (30), allowing to take into account the weight on the results from the evaluation of unit setpoint and input disturbance step responses. As constraints in the optimization, the admissible shape-related constraints have been specified by the vector $\epsilon_{yw},\;\epsilon_{yi},\;\epsilon_{uw},\;\epsilon_{ui}$ as:

(44) $$\begin{array}{c} TV_{0}\left(y_{w}\right)\leq \epsilon_{yw}\;;\;\;TV_{1}\left(y_{i}\right)\leq \epsilon_{yi}\\ TV_{1}\left(u_{w}\right)\leq \epsilon_{uw}\;;\;\;TV_{1}\left(u_{i}\right)\leq \epsilon_{ui} \end{array}$$

Since the above analytical approach studied in Section Äľ yields ideal shapes of transient responses, it can be characterized by constraints:

(45) $$\epsilon =\epsilon_{yw}=\epsilon_{yi}=\epsilon_{uw}=\epsilon_{ui}\to 0$$

In practical applications, each of the permissible restrictions may be specified by a separate number. However, if we want to show general trends in this work regarding the impact of permissible restrictions, we will choose, for simplicity, a vector of ”sufficiently” small figures:

(46) $$\epsilon =\epsilon_{yw}=\epsilon_{yi}=\epsilon_{uw}=\epsilon_{ui}\in \left\{0.001,0.01,0.1\right\}$$

This choice is surely of an ”ad hoc” nature, has a strong influence on the resulting transients, but represents a trade-off between practical usability and computational effort.

In a similar way as in [38], the following theorem can be derived for working with dimensionless quantities and quantities of a real circuit corresponding to the model (4) with $T_{m}=T_{d}$.

**Theorem** **1.** *Let us consider PP, including items $\bar{IAE}\left({\bar{y}}_{s}\right)$, $\bar{IAE}\left({\bar{y}}_{i}\right)$, ${\bar{TV}}_{0}\left({\bar{y}}_{s}\right)$, ${\bar{TV}}_{1}\left({\bar{y}}_{i}\right)$, ${\bar{TV}}_{2}\left({\bar{u}}_{s}\right)$, and ${\bar{TV}}_{2}\left({\bar{u}}_{i}\right)$ generated for the IPDT plant with $K_{s}=1,T_{d}=1$ over the chosen grid of points $K_{c},T_{i},T_{D},b$ by simulating setpoint responses ($w=1,d_{i}=0$) with output and input ${\bar{y}}_{s}\left(\tau \right)$, ${\bar{u}}_{s}\left(\tau \right)$ and input disturbance responses ($w=0,d_{i}=1$) with ${\bar{y}}_{i}\left(\tau \right),{\bar{u}}_{i}\left(\tau \right)$. The properties of these responses are stored and expressed over a grid of dimensionless variables:*

(47) $$\begin{array}{c} \kappa =K_{c}K_{s}T_{d}^{2}\in \left[\kappa_{min},\kappa_{max}\right]\;;\;\;\delta =K_{d}K_{s}T_{d}\in \left[\delta_{min},\delta_{max}\right];\\ \tau_{i}=T_{i}/T_{d}\in \left[\tau_{imin},\tau_{imax}\right]\;;\;\;\;b\in \left[b_{min},b_{max}\right]. \end{array}$$

*Then, the PP items corresponding to any plant model parameters $K_{s},T_{d}$, and prefilter tuning b belonging to the range (47) with the corresponding responses $y_{s}\left(t\right),u_{s}\left(t\right)$, $y_{i}\left(t\right)$, and $u_{i}\left(t\right)$ can be calculated according to:*

(48) $$\begin{array}{c} IAE\left(y_{s}\right)=T_{d}\;\bar{IAE}\left({\bar{y}}_{s}\right)\;;\;\;IAE\left(y_{i}\right)=K_{s}T_{d}^{3}\;\bar{IAE}\left({\bar{y}}_{i}\right)\\ TV_{0}\left(y_{s}\right)={\bar{TV}}_{0}\left({\bar{y}}_{s}\right)\;;\;\;TV_{1}\left(y_{i}\right)=K_{s}T_{d}^{2}\;\;{\bar{TV}}_{1}\left({\bar{y}}_{i}\right)\\ TV_{2}\left(u_{s}\right)=\frac{1}{K_{s}T_{d}^{2}}{\bar{TV}}_{2}\left({\bar{u}}_{s}\right)\;;\;\;TV_{2}\left(u_{i}\right)={\bar{TV}}_{2}\left({\bar{u}}_{i}\right) \end{array}$$

This theorem shows that it is enough to carry out the optimal design just in the space of dimensionless parameters and to calculate the actual values of the performance measures just when wishing to show the relative character of some chosen admissible shape-related deviations (44)–(46), which have to be interpreted in the context of particular model parameters $T_{d}$ and $K_{s}$.

For $s_{w}=0.5$ and $s_{i}=1-s_{w}=0.5$, the search over the stored PP with performance measures satisfying the above requirements yields vectors of optimal controller tuning parameters:

(49) $$\begin{array}{c} \epsilon =0.001:\;\;\kappa_{o}=0.19;\;\delta_{o}=0.6;\;\tau_{D}=3.16;\;\tau_{io}=8;\;b=0.3;\\ \epsilon =0.01:\;\;\kappa_{o}=0.21;\;\delta_{o}=0.66;\;\tau_{D}=3.14;\;\tau_{io}=8;\;b=0.3\\ \epsilon =0.1:\;\;\kappa_{o}=0.24;\;\delta_{o}=0.72;\;\tau_{D}=3.00;\;\tau_{io}=8;\;b=0.5. \end{array}$$

**Figure 6:**
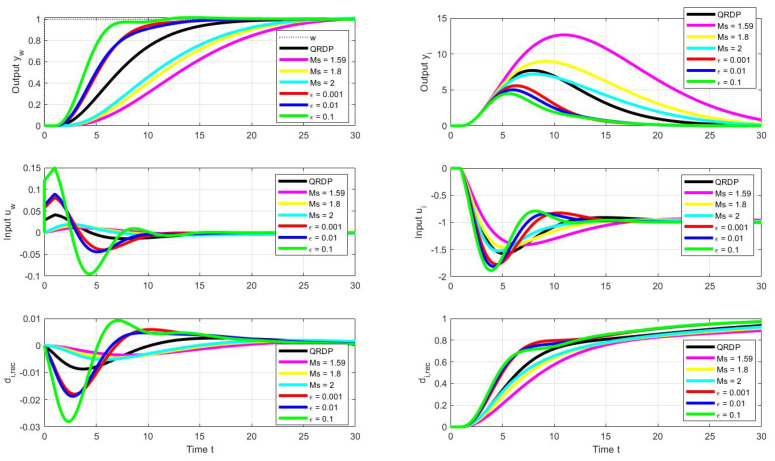
Unit setpoint step responses (**left**) and unit input disturbance step responses (**right**) achieved with the QRDP PID controller (black), responses corresponding to POI-PID controllers [44] with a prescribed $M_{s}$ constraints and responses corresponding to optimal tuning calculated by the PPM with the parameter grid (41) under the performance specifications (46) and (44); $T_{m}=1$; $K_{s}=1$; $T_{s}=0.001$.

A comparison of transients with the QRDP analytical method in Figure 6 shows that PPM makes it possible to find faster transients. Thereby, the advantage of QRDP tuning (24) is that it works with lower controller gains (which is important in noisy loops) and, nominally, it gives ideal response shapes (ϵ=0) for any values of the model parameters $K_{s}$ and $T_{d}$. The non-zero shape deviations specified for dimensionless quantities are subject to changes depending on the parameters of the model when moving to real values of the shape deviations, which means that they can ultimately be significantly different [28,38]. This transformation must be taken into account when specifying search constraints. The speed of transients can be increased by tolerating larger deviations from ideal courses (i.e., by increasing the ϵ values).

**Figure 7:**
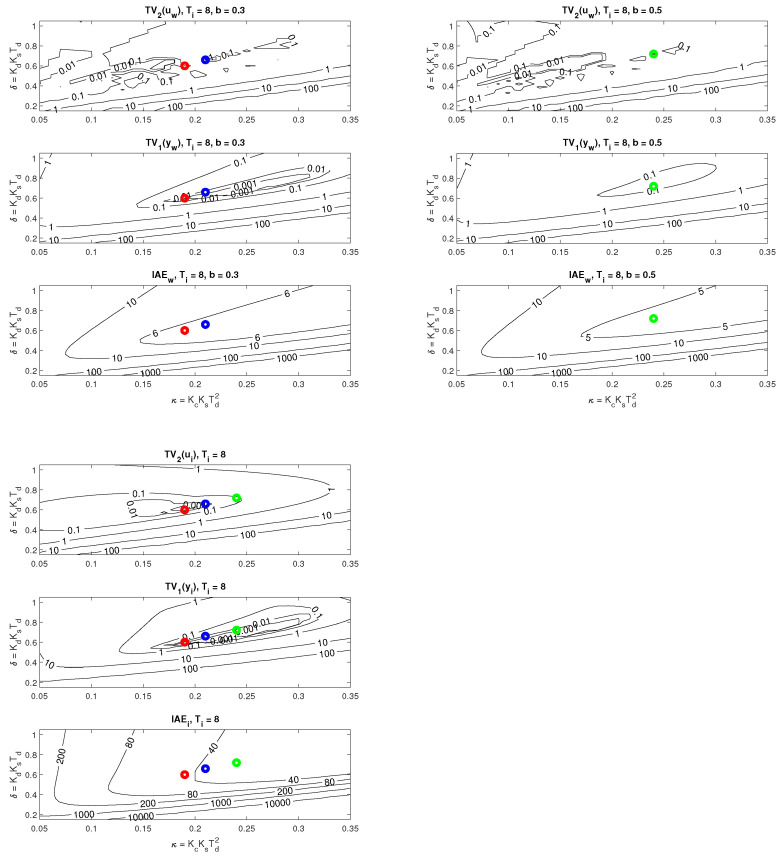
Cross-sections of PP (41) corresponding to setpoint responses (above) and disturbance responses (below) from Figure 6 for the optimal parameters (49) fulfilling the constraints (46) with ϵ=0.001 (red), ϵ=0.01 (blue), and ϵ=0.1 (green).

The location of the found optimal settings in the overall context of PP can be seen using the cross-sections in Figure 7. In the case of setpoint responses, it can be noticed that at higher values of *b*, the area corresponding to the transients with small deviations from the monotonicity disappears. Allowing larger deviations from ideal shapes of transients leads to acceleration (decreased IAE values) of both setpoint and disturbance responses.

**Remark** **3** (Impact of increased loop inertia on the ideal controller output shape). *Allowable areas specified by $TV_{2}\left(u\right)\leq 0.001$ in Figure 7 are very small and difficult to display using contours. However, from the application of Feldbaum’s theorem to a double integrator supplemented by the integral action of the controller and delays, which further increase the circuit order, the question arises whether it might not be interesting to look for solutions with higher allowable $TV_{2}\left(u\right)$ values or directly with higher number of allowable pulses of optimal system input, evaluated, for example, by $TV_{2}\left(u\right)$ [50].*

### 4.3. Parallel PID Controller Tuning Optimized under Consideration of Sensitivity Constraints

To get a picture of the differences in the approaches of traditional PID controller design, often based on IAE optimization supplemented by sensitivity constraints, the responses obtained using PPM and QRDP analytical tuning are compared with the optimal solutions from [44] given for three different $M_{s}$ values:

(50) $$\begin{array}{c} \kappa =0.0694;\;\tau_{D}=5.7675;\;\tau_{i}=13.3862;\;M_{s}=1.59\\ \kappa =0.0974;\;\tau_{D}=5.0837;\;\tau_{i}=11.9603;\;M_{s}=1.80\\ \kappa =0.1215;\;\tau_{D}=4.6796;\;\tau_{i}=11.2708;\;M_{s}=2.00 \end{array}$$

To avoid overshooting of the setpoint step responses, the prefilter (42) with the numerator parameter b=0 is used, whereas the output derivative signal is taken from the plant model. The corresponding transients in Figure 6 show these responses yet much slower than for the QRDP tuning. If the reason for slowing down the responses should be the argument that it is necessary to increase the robustness in case of uncertainty of the model, the answer is that in that case, it is possible to search for other controller tuning using PPM. However, it does not make sense every time, even if the parameters of the model are supposed to be known well enough.

### 4.4. Example 4: Optimal Nominal Tuning of the Series PID Controller

It is well known (see for example [33,44]) that higher dynamics can be achieved with parallel PID controllers than with series controllers. As mentioned in Remark 2, even a parallel QRDP PID controller cannot be converted to an equivalent series controller. However, the parallel controllers only contain the disturbance observer implicitly [27,28]. If we need to explicitly monitor the disturbance for some reason, the controller must be supplemented with an additional block of low-pass filter (as in Figure 5) with the time constant $T_{i}$. From the point of view of disturbance reconstruction, but also with regard to the impact of control signal limitations, it is therefore more advantageous to work with a series PID controller, including an explicit disturbance observer.

Although the specification of the PP (41) calculation was designed with regard to the calculation of the parallel controller, the obtained PP can also be used to calculate the parameters of the series PID controller. At the same time, it can be expected that the fastest achievable transients will correspond to the borderline case in terms of parameter conversion (20), when:

(51) $$T_{ip}=4T_{Dp};\;K_{cs}=K_{cp}/2;\;T_{is}=T_{Ds}=T_{ip}/2.$$

When we search in PP (41) using these constraints, we find that for $s_{w}=0.5$ and $s_{i}=1-s_{w}=0.5$, it is not possible to find an optimal series PID controller that would provide transients with a deviation from ideal shapes ϵ≤0.001.

**Remark** **4** (Influence of rounding errors). *We have already pointed out the small areas of permissible $TV_{2}\left(u\right)$ values in Remark 3. Due to κ and δ quantization in the $\tau_{D}=\delta /\kappa$ calculation and in further conversion steps, the situation will worsen further. The problems could be eliminated by generating another PP for the series PID design, which would work directly with $\kappa ,\tau_{D},\tau_{i}$, and $b_{s}$ corresponding to the prefilter:*

(52) $$F_{ps}\left(s\right)=\frac{bT_{is}s+1}{T_{is}s+1};\;T_{is}=T_{ip}/2$$

*However, it is also possible to use the PP calculated for the parallel PID, but the values of the permissible values of $TV_{2}\left(u\right)$ need to be increased slightly.*

**Figure 8:**
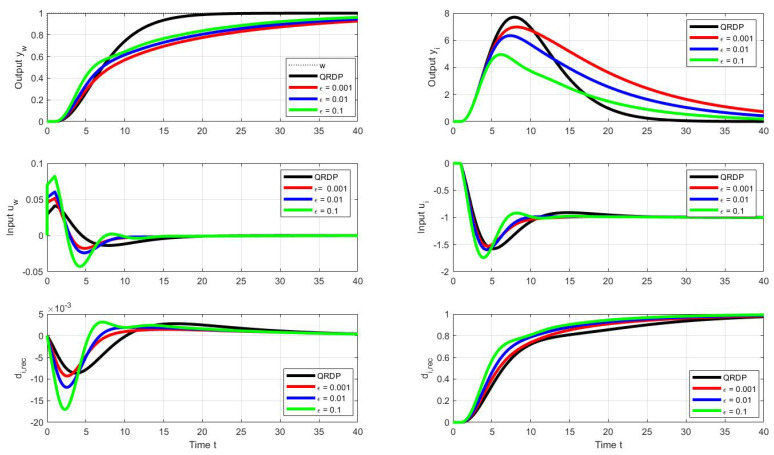
Unit setpoint step responses (**left**) and unit input disturbance step responses (**right**) achieved with the parallel QRDP PID controller (black) and responses corresponding to series PID controller (see Figure 9) with the optimal tuning calculated by the PPM over the parameter grid (41) under the performance specifications (53) and (44); $T_{m}=1$; $K_{s}=1$; $T_{s}=0.001$.

Figure 8, therefore, shows the optimal transients corresponding to the optimal series PID when selecting a modified vector of admissible shape deviations:

(53) $$\epsilon =\epsilon_{yw}=\epsilon_{yi}=\epsilon_{uw}=\epsilon_{ui}\in \left\{0.002,0.01,0.1\right\}$$

Then, the search over the stored PP with performance measures satisfying the above requirements yields vectors of optimal controller tuning parameters (the indices “*p*” and “*s*” are used to distinguish the primarily found dimensionless values of the parallel controller and the recalculated values of the serial PID):

(54) $$\begin{array}{c} \epsilon =0.002:\;\;\kappa_{p}=0.13;\;\delta_{p}=0.57;\;\tau_{ip}=18;\;\kappa_{s}=0.065;\;\tau_{Ds}=9;\;\tau_{is}=9;\;b_{p}=0.7;\\ \epsilon =0.01:\;\;\kappa_{p}=0.15;\;\delta_{p}=0.60;\;\tau_{ip}=16;\;\kappa_{s}=0.075;\;\tau_{Ds}=8;\;\tau_{is}=8;\;b_{p}=0.7;\\ \epsilon =0.1:\;\;\kappa_{p}=0.20;\;\delta_{p}=0.69;\;\tau_{ip}=14;\;\kappa_{s}=0.100;\;\tau_{Ds}=7;\;\tau_{is}=7;\;b_{p}=0.7. \end{array}$$

**Figure 9:**
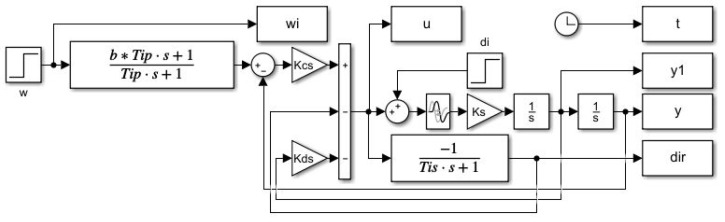
Matlab/Simulink simulation scheme with series PID controller used to verify controller tuning calculated from the PP (41) derived for the parallel PID control; the prefilter $F_{p}\left(s\right)=$$\left(1+bT_{ip}s\right)/\left(1+T_{ip}s\right)$ (42) still corresponds to the parallel PID tuning.

The responses were obtained using a series PID scheme with the output derivative available in Figure 9.

**Figure 10:**
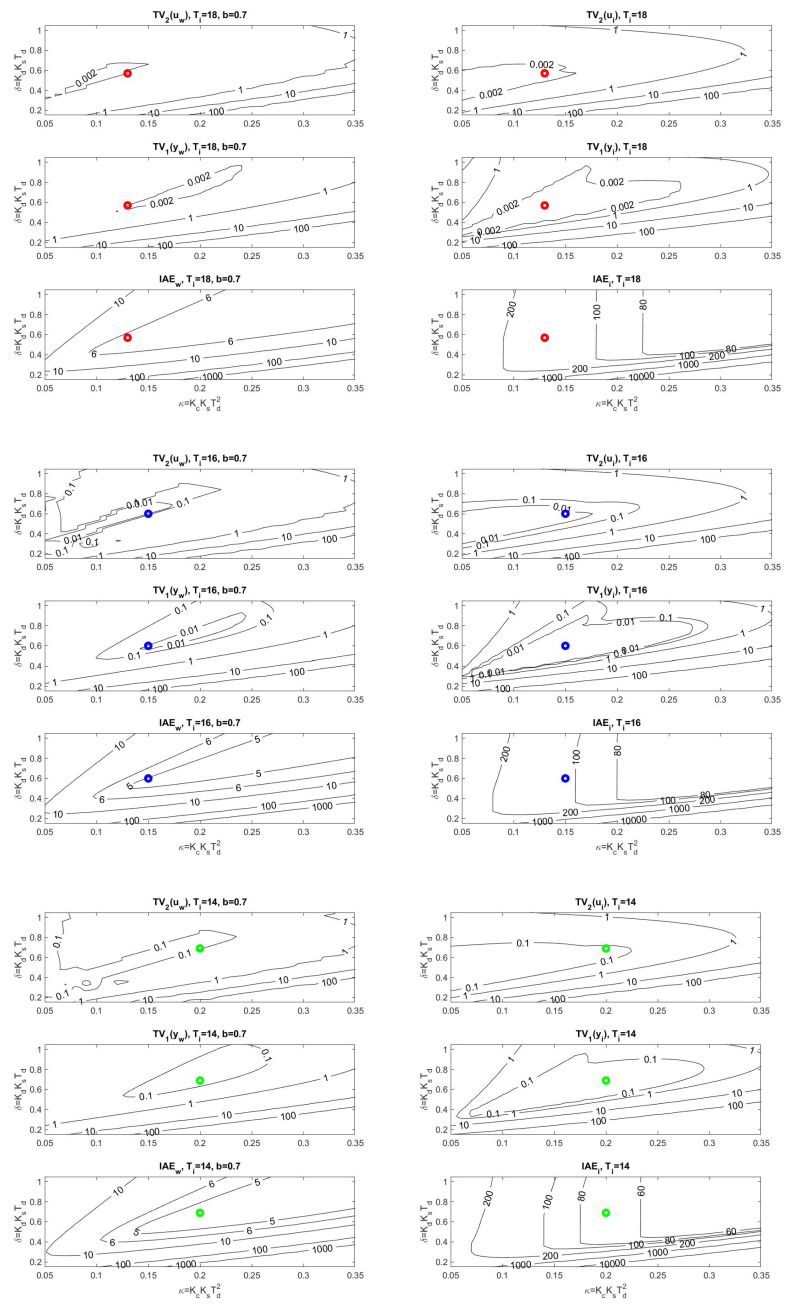
Cross-sections of PP (41) corresponding to setpoint responses (left) and disturbance responses (right) from Figure 8 for the optimal parameters (54) fulfilling the constraints (53) with ϵ=0.002 (red, above), ϵ=0.01 (blue, middle), and ϵ=0.1 (green, below).

A detailed look at the location of the corresponding operating points in the PP (see Figure 10) shows that, in terms of the size of the dimensionless gains, influencing strongly the speed of transients, the limits on the permissible value of $TV_{2}\left(u\right)$ are decisive.

If, in order to accelerate the transients, we keep the allowable shape limits of the output $\epsilon_{yw}$ and $\epsilon_{yi}$ according to (53) and increase the allowable input (controller output) restrictions in all three cases to the value:

(55) $$\epsilon_{u}=\epsilon_{uw}=\epsilon_{ui}=0.3.$$

Another way to accelerate transients would be to accept transients with a higher number of monotonic control intervals at the controller output.

**Figure 11:**
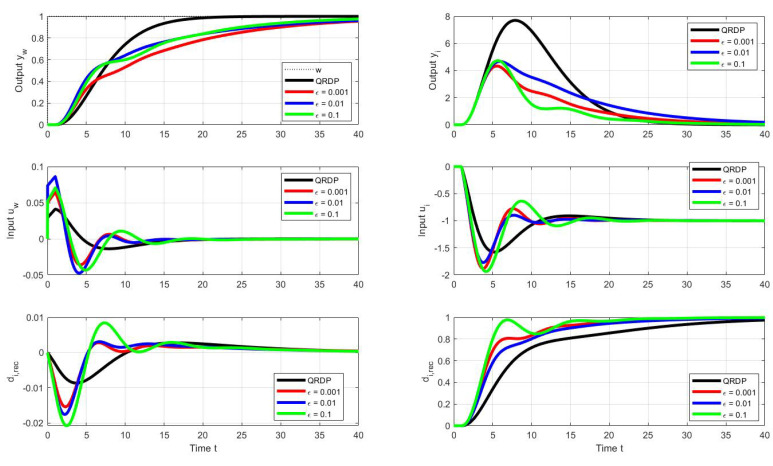
Unit setpoint step responses (**left**) and unit input disturbance step responses (**right**) achieved with the parallel QRDP PID controller (black) and responses corresponding to series PID controller (see Figure 9) with the optimal tuning calculated by the PPM over the parameter grid (41) under the output performance specifications (53) and (44) and the input admissible deviations (55); $T_{m}=1$; $K_{s}=1$; $T_{s}=0.001$.

The resulting waveforms of the individual circuit responses are presented in Figure 11. Even without numerical quantification of the obtained responses, there is an evident significant increase in speed, especially in terms of compensation of disturbance steps, but also in terms of the speed of reconstruction of the input disturbance. Since this can already be noticed in the series PID controller in Figure 9 compared to the reactions of the parallel PID controller Figure 5, it can be stated that while the parallel PID controller allows faster transients in terms of output, a series PID controller with an explicit disturbance observer is faster in terms of disturbance reconstruction.

**Remark** **5** (Limitations on $T_{i}$ selection). *Understanding PID functionality, which is based on disturbance reconstruction from the controller output steady-state values, is crucial to understanding why it is not possible to arbitrarily reduce the integral time constant $T_{i}$ with respect to the transients of the loop stabilised by the PD controller. The existence of $T_{i}$ size limitations, mentioned already in Remark 1, is obvious from all the above analytical and numerical calculations of the optimal PID controller settings. Therefore, countless attempts to overcome this handicap by applying AI methods cannot be considered intelligent.*

### 4.5. Example 5: Considering the Pareto-Front in Setpoint Tracking and Disturbance Rejection

It is well known that a PID controller designed with the best setpoint tracking in mind may not provide optimal disturbance rejection and vice versa [33,38,51]. Depending on the specific requirements of the considered application, it may therefore be interesting to look for the optimal setting of the controller at variable values of the weight parameters $s_{w}$ and $s_{i}=1-s_{w}$ of the cost function (30).

The admissible shape deviations will again be chosen as a mix of the allowable shape limits of the output $\epsilon_{yw}=\epsilon_{yi}=0.001$ and the allowable input deviations (55) with $\epsilon_{uw}=\epsilon_{ui}=0.3$. PP search with $s_{w}=1$ and $s_{w}=0$, whereby $s_{i}=1-s_{w}$, then provides controller parameters in Table 2.

**Figure 12:**
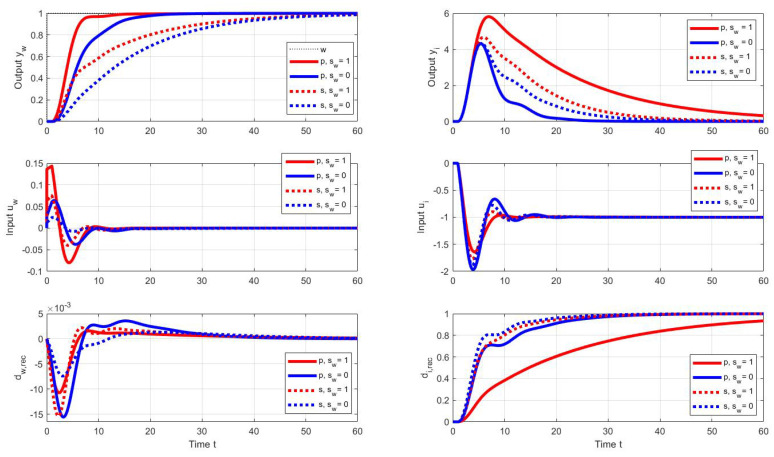
Unit setpoint step responses (**left**) and unit input disturbance step responses (**right**) achieved with the parallel (p) and series (s) PID controllers (black) with the optimal tuning calculated by the PPM over the parameter grid (41) under the output performance specifications (55) and (44) and the cost function weights $s_{w}=1$ and $s_{w}=0$, $s_{i}=1-s_{w}$; $T_{m}=1$; $K_{s}=1$; $T_{s}=0.001$.

The setpoint and disturbance step responses corresponding to the specified parallel and series PID controllers are in Figure 12. As expected, with both controllers, setpoint step responses with $s_{w}=1$ are faster than with $s_{w}=0$. Here, too, the input disturbance is reconstructed more quickly in the case of series PIDs. However, it may come as a surprise that the optimal parallel PID with $s_{w}=1$ has a significantly worse disturbance response than both series PID controllers.

## 5. Discussion

In terms of the need for the laborious initial generation of PP, required for its further use in the design of controllers, PPM represents an approach with high initial computational demands. However, the control design is, by nature, an interactive activity in which we frequently step-by-step strive to achieve the best possible performance by taking into account the often conflicting requirements arising from an infinitely diverse range of demands for real applications. Furthermore, with regard to very simple optimization options based on searching the stored properties of the waveforms corresponding to the individual settings, this is extremely advantageous with the use of PPM. Moreover, this method is also advantageous in terms of the support provided in improving the features of interactive designs (for example, it shows which constraints are dominant in the effort to improve dynamics). In terms of time savings compared to traditional optimization, whose processes must be repeated for each change in the cost function and given constraints, it is significantly more advantageous for interactive PPM design.

We have mentioned several times that the DOB contained in PID controllers is based on the properties of ultra-local integral models of the system. However, we will return to their importance once again. In the design, we try to approximate the practically unlimited range of various dynamical systems using suitably selected mathematical models, for which we implement the design of optional parameters of the controller. From the point of view of the universal applicability of the results achieved by means of individual models, ultra-local integral models [38,56] have proved to be important since the first known method [57] for setting controllers. Although over time it has become possible to achieve a significant improvement in the dynamic properties of the circuit with more complex structures (as, for example, [58]), due to their simplicity, PID controllers still maintain an exceptional place in practice. As mentioned in Section 2, in [33,34,35,44], and in many other ADRC [31,32] or MFC publications [29,30], the use of ultra-local models to approximate different dynamical processes is an important robust control tool in which the system’s internal feedback is included in an equivalent disturbance compensated by an integral action of the controller. Their optimal setting for the control of DIPDT models using PPM thus naturally complements the work [38,39] devoted to the optimal and robust setting of PI controllers for IPDT models.

The digitization of the automatic control design, which is the mainstay of the Industry 4.0–5.0 campaigns, is of course also reflected in the design of the controllers themselves. From this point of view, it is far more advantageous to work with various computer-support systems of the PPM type than with traditional text overviews of optimal settings of [59] controllers. Although analytical methods as [33,49] provide a free parameter to take into account application requirements, the impact of that parameter may not be predictable enough to allow effective design. The optimization method with sensitivity constraints are, in general, not effectively and consistently applicable to unstable systems.

Regarding the development trends in the field of automatic control, we should note the increase in open solutions, in which the designer does not have to rely on a relatively narrow offer of industrially manufactured controllers with a relatively fixed structure, but implements its own solution using a far more universal base of programmable embedded controllers. This is also related to the development of ever new regulators and structures [58,60], which significantly push the boundaries of achievable circuit characteristics. It follows from this statement that the PPM will also need to be developed for the design of additional controllers with more advanced and efficient disturbance observers.

## 6. Conclusions

The paper discussed the optimal design of series and parallel PID controllers for a double integrator plus dead-time process using the performance portrait method. The proposed design imitates an expert who selects the most suitable solution based on appropriate criteria (performance measures) from a database of known solutions. The database of relevant responses, referred to as the “performance portrait”, is evaluated in terms of the weighted sum of the IAE criterion in closed-loop tracking and disturbance rejection, and the shape of the input and output signals of the process (compared to their ideal shapes). This new intelligent control method has no convergence problems and allows interactive multi-criteria optimization of the control according to the required performance and robustness.

## Figures and Tables

**Table 1:** Performance measures corresponding to the setpoint step responses in Figure 4.

-	QRDP	TRDPb	TRDP0	Gerov-Jovanovic [45]
$IAE_{w}$	9.8868	5.1608	7.2944	5.4013
$TV_{0}\left(y_{w}\right)$	0.0000	0.0000	0.0000	0.0457
$TV_{2}\left(u_{w}\right)$	0.0000	0.0000	0.0000	0.0033

**Table 2:** Parameters of parallel (pPID) and series PID controllers (sPID) found for $\epsilon_{yw}=\epsilon_{yi}=0.001$ and $\epsilon_{uw}=\epsilon_{ui}=0.3$ in PP (41); $\kappa_{p}=K_{s}K_{cp}T_{d}^{2}$, $\delta_{p}=K_{s}K_{dp}T_{d}$, $\tau_{ip}=T_{ip}/T_{d}$, $b_{p}$, $\kappa_{s}=\kappa_{p}/2$, $\tau_{is}=\tau_{ip}/2$ and $\tau_{Ds}=\tau_{ip}/2$.

2DoFPID	$s_{w}$	$\kappa_{p}$	$\delta_{p}$	$\tau_{\mathrm{ip}}$	$b_{p}$	$\kappa_{s}$	$\tau_{\mathrm{is}}$	$\tau_{\mathrm{Ds}}$
Parallel	1	0.1700	0.6300	22	0.8	-	-	-
	0	0.2700	0.7200	8	0.1	-	-	-
Series	1	0.2100	0.7200	14	0.6	0.1050	7	7
	0	0.2500	0.7500	12	0.1	0.1250	6	6

## Data Availability

Not applicable.

## Abbreviations

The following abbreviations are used in this manuscript:

1P	One-Pulse, response with 2 monotonic segments (1 extreme point)
2P	Two-Pulse, response with 3 monotonic segments (2 extreme points)
3P	Three-Pulse, response with 4 monotonic segments (3 extreme points)
3D	Three-Dimensional
ADRC	Active Disturbance Rejection Control
AI	Artificial Intelligence
DIPDT	Double Integrator Plus Dead-Time
IAE	Integral Absolute Error
IPDT	Integrator Plus Dead-Time
MFC	Model-Free Control
MFRP	Modified sets of Four Real Poles
MRDP	Multiple Real Dominant Pole
PD	Proportional-Derivative
PID	Proportional-Integral-Derivative
PP	Performance Portrait
PPM	Performance Portrait Method
QRDP	Quadruple Real Dominant Pole
TRDP	Triple Real Dominant Pole
TV	Total Variation
$TV_{0}$	Deviation from Monotonicity
$TV_{1}$	Deviation from 1P Shape
$TV_{2}$	Deviation from 2P Shape
$TV_{3}$	Deviation from 3P Shape
